# Heart in Focus: Advancing Pericardial Effusion Diagnosis With Point-of-Care Ultrasound

**DOI:** 10.7759/cureus.76681

**Published:** 2024-12-31

**Authors:** Sofia Moura de Azevedo, Rodrigo Duarte, Jéssica Krowicki, Dolores Vázquez, Sheila Pires Ferreira Arroja, José Mariz

**Affiliations:** 1 Internal Medicine, Centro Hospitalar Universitário de Santo António, Porto, PRT; 2 Internal Medicine, Centro Hospitalar de Lisboa Ocidental, Lisbon, PRT; 3 Internal Medicine, Centro Hospitalar do Baixo Vouga, Aveiro, PRT; 4 Emergency, Hospital de Braga, Braga, PRT

**Keywords:** echocardiography - pericardial - effusion, emergency pericardiocentesis, pericardial diseases, pericardial tamponde, point-of-care ultrasound (pocus)

## Abstract

Pericardial effusion refers to the accumulation of fluid within the pericardial sac, the double-layered membrane surrounding the heart. It can be caused by various medical conditions and may lead to serious complications if not diagnosed and managed promptly. Point-of-care ultrasound (POCUS) has emerged as a valuable tool in the clinical evaluation of pericardial effusions, offering real-time visualization and aiding in the assessment of its size, characteristics, and potential hemodynamic impact.

This comprehensive revision explores the utility of POCUS in diagnosing and managing pericardial effusions. POCUS has gained prominence as a bedside diagnostic tool due to its immediacy, accuracy, and non-invasive nature. This study investigates how POCUS can address critical gaps in current diagnostic approaches, such as delays in diagnosis using traditional imaging modalities and challenges in resource-limited settings, thereby enhancing patient outcomes and clinical decision-making.

A search was conducted on PubMed in August of 2023, using the keywords "POCUS" and "pericardial" as MeSH terms and reference mining. A total of 19 articles were included in this review.

Characterization and quantification of pericardial effusion (PEF) using POCUS can provide clinicians with critical clues regarding the underlying etiology. This information, combined with other hemodynamic parameters, should guide subsequent management decisions. POCUS enables the identification of key sonographic findings, such as diastolic collapse of the right chambers, abnormal septal movement, and an engorged inferior vena cava (IVC), which together raise a high clinical suspicion of cardiac tamponade. Beyond its utility in identifying tamponade, POCUS plays a significant role in detecting subtle yet life-threatening conditions, such as aortic dissection, which may manifest as pericardial effusion due to hemopericardium. While POCUS is not definitive for diagnosing aortic dissection, indirect findings such as a pericardial effusion with hemodynamic compromise, coupled with high clinical suspicion, should prompt further imaging like CT angiography for confirmation. We propose an algorithmic approach: if cardiac tamponade is confirmed on POCUS, emergent pericardiocentesis is warranted. If ruled out, further investigations should be directed toward identifying the underlying cause of the PEF, including potentially ruling out aortic dissection to avoid missing a subtle but critical condition.

POCUS has revolutionized the clinical evaluation of pericardial effusions, providing clinicians with a rapid and accurate bedside tool for diagnosis and management. Its ability to assess effusion size, identify cardiac tamponade, and guide pericardiocentesis procedures has proven invaluable in improving patient outcomes. Integrating POCUS into routine clinical practice enhances diagnostic accuracy and timely intervention, ensuring better care for patients with pericardial effusions. However, it is important to acknowledge its limitations. POCUS is highly operator-dependent, with diagnostic accuracy varying based on the clinician's experience and training. Additionally, the availability of ultrasound equipment and adequately trained personnel can be a barrier, particularly in resource-limited settings. Addressing these challenges is crucial to maximizing the utility of POCUS in clinical practice.

## Introduction and background

Bedside ultrasound offers significant benefits, such as real-time imaging, non-invasiveness, portability, and quick availability, making it an increasingly valuable tool for assessing medical patients. As the use of point-of-care ultrasound (POCUS) becomes more prevalent in internal medicine, it is important to establish a relevant curriculum for residency training programs. The Canadian Internal Medicine Ultrasound (CIMUS) group has provided consensus-based recommendations for this purpose. They suggest that the core curriculum should include four key POCUS applications: assessing the inferior vena cava (IVC), identifying lung B lines, detecting pleural effusion, and evaluating for abdominal free fluid. Additionally, they recommend training in three ultrasound-guided procedures: central venous catheterization, thoracentesis, and paracentesis [1]. For an expanded curriculum, they propose including seven more POCUS applications, such as internal jugular vein assessment, lung consolidation, pneumothorax detection, knee effusion evaluation, left ventricular systolic function assessment, pericardial effusion detection, and right ventricular strain assessment, along with four additional ultrasound-guided procedures, including knee arthrocentesis, arterial line insertion, arterial blood gas sampling, and peripheral venous catheterization. While the CIMUS framework addresses a range of applications, its relevance to pericardial effusion evaluation is highlighted by its focus on rapid, bedside diagnostics for hemodynamic assessment. Specifically, CIMUS advocates for the integration of cardiac POCUS in internal medicine to detect life-threatening conditions such as cardiac tamponade through real-time visualization of diastolic chamber collapse, abnormal septal motion, and IVC engorgement. These features provide immediate diagnostic information that traditional imaging methods often fail to deliver promptly. POCUS offers significant advantages over other diagnostic methods, such as echocardiography performed in a formal imaging suite or CT imaging, by allowing near-instantaneous assessment at the point of care [1-4].

Cardiac point-of-care ultrasound aims to rule out obvious pathologies that can impact the treatment and clinical outcomes of the patients evaluated. Multiple studies have shown that POCUS is associated with earlier diagnosis and intervention for clinically significant effusions [5-8]. In this review, the focus will be on the evaluation of pericardial effusion (PEF) and signs of cardiac tamponade. While POCUS can also provide valuable information on dimensions of cardiac cavities, wall thickness, and gross left and right ventricular systolic function, these aspects are beyond the scope of this review. Early diagnosis of PEFs through bedside ultrasound allows for the timely initiation of appropriate management. Depending on the clinical context and the severity of the effusion, management options may include observation, medical treatment, or, in cases of cardiac tamponade or significant effusions, drainage of the fluid through pericardiocentesis. In emergency settings, where delays can mean the difference between life and death, POCUS stands out for its speed. POCUS reduces the time to pericardiocentesis, with median times ranging from 11.3 to 28.1 hours, compared to 34.6 to over 48 hours without POCUS [6-8]. POCUS also facilitates faster cardiology-based echocardiography when necessary [5-11]. The diagnostic speed is linked with improved outcomes, being associated with a shorter hospital stay and reduced 28-day mortality rates for patients diagnosed with PEF in the emergency department, compared to those evaluated using delayed or traditional imaging methods [5]. 

## Review

Aim

The aim of this comprehensive review is to highlight the utility and accuracy of POCUS as a valuable tool in clinical practice, particularly in high-stakes settings such as the emergency department (ED) and intensive care unit (ICU), where rapid and accurate decision-making is critical. This review focuses on the effective use of POCUS for the timely diagnosis and management of PEFs and related conditions, such as cardiac tamponade. By addressing existing knowledge gaps, such as the variability in operator expertise and challenges in integrating POCUS into routine workflows, and emphasizing its ability to enhance diagnostic accuracy and speed, the review aims to underscore the life-saving potential of POCUS. Ultimately, this review seeks to contribute to improved patient outcomes by facilitating the early detection and appropriate management of pericardial diseases, even in resource-limited settings.

Methods

A search was conducted on PubMed in August 2023 using the keywords "POCUS" and "pericardial" as MeSH terms. A total of 19 articles were included in this review. These studies encompassed a mix of retrospective cohort studies, observational research, review articles, and expert consensus recommendations. The review focused on literature that examined the diagnostic accuracy, clinical utility, and time-to-intervention benefits of POCUS in detecting and managing pericardial effusions. Reference mining was used to identify additional relevant studies, ensuring comprehensive coverage of the topic. The articles reviewed were published between 2013 and 2023.

Results and discussion

The Role of POCUS in the Diseases of the Pericardium

A PEF is the accumulation of excess fluid in the space between the layers of the pericardium, the pericardial cavity. Under normal circumstances, there is physiological serous fluid inside the pericardial sac, which acts as a lubricant for the heart. A PEF is generally considered when the volume of fluid exceeds 15-50 ml; however, this threshold may vary depending on the literature and clinical context. PEF can have various underlying causes, including pericarditis (idiopathic, infective, cancer-related, autoimmune, uremic), medical procedures (e.g., cardiac intervention), myocardial infarction, thyroid dysfunction, trauma, and acute aortic syndrome. Symptoms of PEF can vary widely, primarily depending on the rate of fluid accumulation. A slow buildup of fluid in the pericardial space may not cause noticeable symptoms, as the pericardium can gradually expand to accommodate the excess fluid. However, when fluid accumulates rapidly, even in small volumes, it can quickly raise pressure within the pericardium. This increased pressure can impair the heart's ability to fill and function properly, potentially leading to a serious condition known as cardiac tamponade. Cardiac tamponade is a life-threatening condition where increased intrapericardial pressure compresses the heart, impairing its ability to effectively pump blood. This results in decreased cardiac output and can lead to a variety of symptoms, such as shortness of breath, chest pain, dizziness, and hypotension. Key diagnostic ultrasound findings play a critical role in identifying cardiac tamponade. These include the diastolic collapse of the right atrium and ventricle, which indicates increased pressure within the pericardial sac. Respiratory variation in mitral or tricuspid inflow velocities can also suggest tamponade physiology, as the heart's ability to fill is restricted during the respiratory cycle. Additionally, an engorged inferior vena cava (IVC), with limited or no respiratory variation, is often seen in tamponade, reflecting impaired venous return to the heart. These ultrasound findings are essential for rapid and accurate diagnosis, especially in emergent clinical settings [9-15]. When PEF is suspected, bedside ultrasound can be performed by a trained healthcare provider, including non-cardiologists, in various clinical settings, including the emergency department, intensive care unit, or at the patient's bedside. Ultrasound, particularly POCUS, is often the first-line examination for diagnosing pericardial diseases due to its accessibility, speed, and ability to provide real-time visualization. In many clinical settings, it can also help infer the etiology of pericardial effusions. Studies support that POCUS reliably detects >95% of PEFs compared to formal echocardiography. While POCUS is highly effective and sensitive in detecting pericardial effusions (PEFs), it is not without limitations, and in specialized or resource-rich environments, traditional echocardiography may still be preferred due to factors such as operator expertise, availability of more advanced equipment, or the need for detailed imaging that POCUS may not fully provide. The accuracy and utility of POCUS are influenced by operator dependency, requiring adequate training and experience to interpret findings correctly and overcome technical challenges. These limitations highlight the importance of continuous operator training to maximize the diagnostic potential of POCUS [9].

Suspecting of Pericardial Effusion 

Finding a pericardial effusion: When performing bedside cardiac ultrasound, there are specific ultrasound signs that indicate the presence of PEF. The pericardium, a double-layered membrane surrounding the heart, appears as a hyper-echoic layer in echocardiography. PEF appears as an area without echoes between the heart and the outer layer of the pericardium on an echocardiogram. This fluid accumulation is typically seen in the dependent regions, such as the posterior, lateral, and inferior walls of the heart. PEFs are commonly visualized using the subxiphoid cardiac view. However, if this view is unavailable, the parasternal long and short-axis views can serve as useful alternatives. To obtain the subxiphoid view, position the ultrasound probe caudal to the sternum, resembling a screwdriver grip. Aim the orientation marker towards the patient's right, and then rotate the probe counterclockwise by 10°-15º. This particular view allows simultaneous visualization of the pericardium and all heart chambers. Deep inspiration is beneficial for imaging, as it enhances venous return and moves the heart towards the ultrasound probe. Depending on the patient’s body build, obtaining this image may require adjusting the depth setting on the ultrasound machine to 20-25 cm to capture the entire heart. This adjustment ensures that the entire pericardial and cardiac structures are visualized, irrespective of the probe's physical position or depth. The parasternal long-axis view is another effective method for detecting pericardial effusions and does not necessitate patient cooperation. To acquire this view, with the on-screen orientation indicator on the left side, position the probe just left of the sternum, within the third or fourth intercostal space (i.e., abdominal mode). Aim the orientation marker toward the patient's left shoulder [8-10]. 

Confirming the finding, be aware of the pitfalls: Several pitfalls and potential sources of misinterpretation should be considered when using echocardiography to diagnose PEF and cardiac tamponade. The key factors affecting fluid visualization and measurement accuracy are body habitus, patient positioning, image quality, and interobserver variability. Each view used to assess PEFs presents specific challenges due to the interplay of anatomical, physiological, and technical factors. These pitfalls often arise from acoustic similarities, interference from adjacent structures, and patient-specific limitations, which can lead to diagnostic challenges. The subxiphoid view is commonly favored in emergencies for its ability to provide a broad perspective of the pericardium. However, it can be particularly challenging in obese patients or those with significant abdominal distention. Acoustic similarities between fluid and fat in obese individuals can obscure visualization, as both appear hypoechoic on ultrasound. Additionally, interference from bowel gas, which scatters ultrasound waves, can further limit clarity. For example, in a patient presenting with hypotension and abdominal distention, gas artifacts may mimic the appearance of a pericardial effusion, delaying the diagnosis of cardiac tamponade. The liver, which serves as an acoustic window in this view, may not transmit sound effectively in patients with hepatic steatosis or hepatomegaly, further obscuring the heart and pericardium. The parasternal long-axis view is critical for differentiating pericardial from pleural effusions and evaluating the descending aorta. However, overlapping anatomical structures and suboptimal acoustic windows caused by chest wall deformities, large breasts, or even incorrect transducer placement can lead to misinterpretations. For instance, a pleural effusion may appear to encroach on the pericardial space, leading to a false-positive diagnosis of a pericardial effusion. Similarly, failure to visualize the descending aorta in its proper plane may obscure subtle findings of aortic dissection, a life-threatening condition. The apical four-chamber view allows for a comprehensive assessment of chamber collapse, but it can be difficult to obtain in patients with significant obesity, where the position of the heart deviates from the typical anatomical orientation. For example, a critically ill patient with an engorged inferior vena cava and hypotension might have subtle right ventricular collapse on this view, which could be misinterpreted as an artifact due to suboptimal probe angulation or interference from lung tissue [2-4,16]. Table 1 outlines common pitfalls encountered during bedside cardiac ultrasound evaluations of the pericardium and provides guidance on how to address them.

**Table 1:** Most frequent pitfalls in bedside cardiac ultrasound evaluation of the pericardium. PEF: pericardial effusion

We should differentiate pericardial fluid from…	What to do
Pleural fluid	Visualizing the descending aorta in the parasternal long-axis view can be helpful. In the case of PEF, the descending aorta remains posterior to the fluid, while in pleural effusion, it lies anterior to the fluid and does not extend beyond the atrioventricular groove. Additionally, incorporating other views, such as lateral chest wall views, can aid in differentiating pleural from pericardial effusions, helping confirm their extra-pericardial location.
Epicardial fat	Epicardial fat is the adipose tissue accumulated between the visceral pericardium and the myocardium. Lies within the pericardial sac, appears as an echogenic structure on the parasternal long-axis view.It is more echogenic than pericardial fluid, which typically appears as a hypoechoic area. A key visual characteristic that helps in differentiating the descending aorta from surrounding fluid is its motion in sync with the myocardium during the cardiac cycle. This movement serves as a distinguishing feature that confirms the structure as the descending aorta, not pericardial fluid. Normally distributed only in the anterior groove, it can be mistaken for an organized PEF. Typical echoic appearance is more commonly found in aged or obese patients. There is evidence of its increasing prevalence as a marker of visceral fat.
Ascites	Ascites below the diaphragm may mimic PEF. Ascites appears invariably in subcostal views, anterior to the right cardiac chambers. The falciform ligament can sometimes be observed within the fluid collection, which, in combination with visible diaphragmatic movement, can help confirm the diagnosis. However, identifying the falciform ligament may not be practical for all users, as it requires a certain level of expertise in ultrasound imaging.​​ In most cases, ascites can be detected in other peritoneal spaces as well.

POCUS Evaluation of a Pericardial Effusion

Characterization and quantification of the pericardial effusion: First, we should take note if it is an unechogenic space, corpusculated or echogenic fluid (Table 2). Additionally, we should assess for the presence of solid masses within the pericardium [2-6]. 

**Table 2:** Categories based on its echogenicity and some considerations.

Echogenicity	Definition	Considerations
Anechoic fluid (unechogenic)	Appears as a dark or black area on ultrasound due to the absence of echoes. This is typical of clear fluid.	Anechoic fluid is typically easier to visualize in healthy patients with normal body habitus. However, obese patients or those with abdominal distension may present challenges in visualizing this fluid clearly due to increased depth or artifact interference.
Echogenic fluid	Appears lighter than anechoic fluid and may contain small particles or fibrin strands, which reflect ultrasound waves.	The presence of echogenic fluid can sometimes complicate the differentiation between fluid and other structures. For instance, it can be mistaken for fat, leading to diagnostic confusion. Identifying the fluid's relationship with the heart structures is crucial for accurate diagnosis.
Complex or corpusculated fluid	Contains echogenic debris or fibrin strands that appear as small particles within the fluid. This fluid may also show motion, which can help distinguish it from non-moving structures.	The echogenicity may change over time as the fluid becomes more organized or if it contains blood. Moving debris or fibrin clots in the fluid can create a shifting appearance, requiring careful monitoring over time.

Then, estimating the size of pericardial effusions is essential in determining the clinical significance and guiding subsequent management. POCUS enables the measurement of the maximal effusion depth in the subcostal, parasternal, and apical views. This information aids in risk stratification and decision-making regarding the need for therapeutic interventions. A PEF refers to the accumulation of fluid in the pericardial space that exceeds 15-50 mL, a threshold above which it may be detected on ultrasound. The size of the effusion can be semi-quantified by measuring the distance between the two pericardial layers, using a standardized approach as outlined in the European Association of Cardiovascular Imaging (EACVI) 2015 recommendations. This measurement is typically taken in the parasternal long-axis view, where the anterior pericardial layer is most visible and accessible. The measurement is made between the pericardial layers at their widest point, around the level of the left ventricle. For global effusion, this measurement should be performed both anteriorly and posteriorly to assess the full extent of fluid accumulation. In some cases, an additional measurement in the apical four-chamber view may be useful to capture effusion around the posterior heart and assess for any asymmetry in fluid distribution. Based on this measurement, we can categorize different degrees of effusion [2-6]. Small effusion: echo-free space in diastole less than 10 mm, corresponding to approximately 300 ml of fluid; moderate effusion: echo-free space in diastole measuring 10-20 mm, corresponding to approximately 500 ml of fluid; large effusion: echo-free space in diastole greater than 20 mm, corresponding to approximately 700 ml of fluid.

The clinical significance of the effusion, such as whether it is mild, moderate, or severe, depends on its volume and the presence of symptoms or hemodynamic effects. The need for intervention is usually determined by clinical signs of tamponade and ultrasound findings. The clinical threshold for intervention generally ranges from moderate to large effusions [2-6].

Assessment of Hemodynamic Impact

Large PEFs can lead to cardiac tamponade, a life-threatening condition characterized by impaired cardiac filling due to increased pericardial pressure. POCUS can provide real-time visualization of cardiac chambers and assist in the evaluation of hemodynamic parameters. We should look for signs of cardiac tamponade, such as right atrial (RA) collapse during ventricular end-diastole, right ventricular (RV) diastolic collapse, indicating elevated intrapericardial pressure, and plethoric inferior vena cava (IVC) with limited respiratory variation. Doppler analysis of tricuspid and mitral flow velocities to assess ventricular interdependence is considered a more advanced technique [2-17].

In the presence of shock and a PEF, we propose a systematic and focused approach of the hemodynamic impact of the PEF by POCUS, based in the pathophysiology of the disease and the risk of tamponade:

Diastolic collapse of cardiac chambers: The diastolic collapse of the right atrium (RA) is a significant echocardiographic finding in cardiac tamponade and is one of the earliest signs of the condition. This occurs during atrial relaxation, when the RA volume is at its minimum and pericardial pressure is at its peak, causing the RA to bow inward. This phenomenon has a high sensitivity for identifying cardiac tamponade, especially when it persists for more than one-third of the cardiac cycle. However, while sensitivity is high, the specificity is moderate, as brief RA collapse can also occur in other conditions, such as elevated intrathoracic pressure. Sustained RA collapse, however, remains a strong indicator of cardiac tamponade. In contrast, right ventricular (RV) diastolic collapse is observed when the RV volume is still low, typically during early diastole. While RV diastolic collapse is less sensitive than RA collapse for detecting tamponade, it is highly specific for the condition when it occurs. However, in certain clinical situations, such as in cases of RV hypertrophy or significantly elevated RV diastolic pressure (e.g., acute or chronic cor pulmonale), collapse of the right heart chambers may not be evident, even in the presence of tamponade. Additionally, regional tamponade, where loculated pericardial effusions or post-surgical adhesions restrict the filling of certain heart chambers, may lead to an incomplete or asymmetric collapse of the right atrium or ventricle, complicating the diagnosis. These situations, particularly with loculated or regional tamponade, require careful attention to the distribution of the effusion and may benefit from serial imaging to assess changes over time, especially when findings are initially ambiguous. In cases where the right heart pressures are elevated or when tamponade findings are not conclusive, serial imaging may be necessary to monitor for progressive changes. Isolated collapse of the left ventricle is rare because the left ventricle’s muscular nature makes it less susceptible to collapse under pericardial pressure unless there is a significant or regional effusion affecting its filling [2-17].

Abnormal movement of the interventricular septum: In pericardial tamponade, the pressure dynamics during the different phases of the cardiac cycle are altered due to the compression of the heart by the accumulated fluid in the pericardial cavity. This results in decreased ventricular filling and stroke volume. During inspiration, the pressure drop at the pericardial level is less compared to the intrathoracic level, leading to a reduced left ventricular filling gradient. This reduction in left-sided cardiac filling causes the ventricular septum to shift towards the left, which in turn increases right ventricular filling. During expiration, the septum shifts back to the right. This dynamic shift of the ventricular septum, commonly referred to as 'septal bounce' or 'ventricular interdependence,' is a hallmark of pericardial tamponade. It occurs due to the altered pressure dynamics caused by the accumulation of fluid in the pericardial cavity. Septal bounce can also be visualized using two-dimensional M-mode echocardiography. However, it is important to note that ventricular interdependence is not exclusive to tamponade and can occur in other conditions, such as chronic obstructive pulmonary disease, acute pulmonary embolism, or conditions involving right or left ventricular failure. In these scenarios, the interdependence is due to elevated pressures or altered filling dynamics in one ventricle affecting the other, although the severity and pattern of septal motion can differ from what is seen in tamponade. The degree of septal shift correlates with the severity of tamponade; a more pronounced shift is typically seen in cases of severe tamponade [2-17].

Dilated aortic root and a flap in the ascending aorta: The defining ultrasound feature of aortic dissection is the detection of an intimal flap, visible as a thin, echogenic line separating the true and false lumens within the aorta. This flap is most easily identified in the anterior portion of the descending aorta using both parasternal long-axis and apical four-chamber views. The proximal ascending aorta can be assessed through the parasternal long-axis view. Additional findings that may support the diagnosis include enlargement of the aortic root (more than 4 cm), aortic regurgitation, and the presence of pericardial effusion. However, transthoracic echocardiography (TTE) has limitations in evaluating the distal ascending aorta and the arch, where the dissection flap may not be well visualized due to shadowing or acoustic window limitations. In such cases, transesophageal echocardiography (TEE) or computed tomography angiography (CTA) may be more effective, providing better visualization of the aorta and aortic arch. Aortic root dilation can be a critical sign not only in the context of dissection but also in underlying conditions like connective tissue disorders (e.g., Marfan syndrome) and chronic hypertension, where long-standing pressure overload can lead to structural changes. For optimal imaging of the aortic root and intimal flap, adjustments in patient positioning may be necessary (e.g., left lateral decubitus position or altering probe angle), and in some cases, higher-frequency transducers may enhance resolution, especially for detailed visualization of the aortic root. The sensitivity of TTE for diagnosing type A aortic dissection ranges from 78% to 90%, with specificity between 87% and 96%. While TTE is often used as the initial diagnostic tool, its limitations in evaluating the full extent of aortic dissection underline the importance of confirming the diagnosis with advanced imaging techniques, particularly for detailed visualization of the aortic arch and distal ascending aorta [2-17].

Plethoric inferior vena cava: During normal breathing, inspiration generates negative intrathoracic pressure, leading to increased venous return from the inferior vena cava (IVC) to the RA. However, in cases of pericardial tamponade, the RA is unable to accommodate the incoming preload due to compression from increased intrapericardial pressure. As a result, the IVC remains plethoric, with minimal respiratory variation. This characteristic sign serves as a highly sensitive indicator for tamponade (95-97%), with a valuable negative predictive value. However, it has lower specificity (~40%) and can also be observed in conditions such as chronic lung disease, congestive heart failure (CHF), tricuspid regurgitation, and other cardiac conditions. Visualization of the plethoric IVC is typically achieved from the sagittal plane below the xiphoid process. The diameter is measured approximately 2-3 cm from the IVC-RA junction, around the level of the hepatic vein draining into the IVC. In some cases, M-mode can be used to obtain specific diameter measurements by placing the cursor through this point. A plethoric IVC is typically defined as having a diameter greater than 2.1 cm with less than 50% inspiratory reduction. However, experienced sonographers can often visually assess the plethoric nature of the IVC without the need for precise measurements. While IVC assessment is a key sign, conditions such as pregnancy or intra-abdominal hypertension can result in false positives, leading to an engorged IVC despite normal right atrial pressures. Conversely, conditions such as hypovolemia, intra-abdominal pressure increases, or severe tricuspid regurgitation can cause the IVC to collapse, potentially masking signs of tamponade. To enhance the reliability of IVC assessment, complementary findings, such as hepatic vein Doppler patterns, can be helpful. In tamponade, hepatic vein flow may demonstrate diminished respiratory variation due to reduced venous return, while in conditions like CHF or pulmonary hypertension, hepatic vein flow may exhibit more consistent variations due to altered pressures. Sonographer experience plays a crucial role in the accuracy of IVC assessment. While quantitative measurement provides a more objective evaluation, experienced sonographers may rely on visual assessment for plethoric IVC, recognizing patterns that correspond with tamponade [18].

In Table 3, the authors offer a further systematic overview of findings in bedside cardiac ultrasound and their importance.

**Table 3:** Systematized findings in bedside cardiac ultrasound and their importance.

Findings not to dismiss	Their importance
Right chambers diastolic collapse	High sensitivity and specificity for identifying cardiac tamponade, especially when it persists for more than one-third of the cardiac cycle.
Abnormal movement of the septum	Additionally confirms altered pressure dynamics.
Dilation of aortic root and intimal flap	Intimal flap is a pathognomonic sonographic feature of aortic dissection.
Plethoric inferior vena cava	If collapsed, points to exclude cardiac tamponade.

The pathologically enhanced ventricular interdependence leads to characteristic respiratory changes in cardiac chamber filling. Doppler echocardiography can complement these findings by assessing flow velocities across the mitral and tricuspid valves. The mitral inflow velocity typically decreases during inspiration due to reduced left ventricular filling, while the tricuspid inflow velocity increases as a compensatory mechanism for enhanced right ventricular filling. The inspiratory increase in RV inflow velocity is greater than 35-40%, and there is a parallel reduction in LV inflow and aortic ejection velocities (although slightly delayed for transpulmonary transit). These Doppler velocity changes provide further insight into the altered hemodynamics associated with cardiac tamponade. But, when using Doppler ultrasound to assess for pericardial tamponade, it is essential to choose the appropriate modality and site based on the clinical context. Pulsed-wave Doppler (PWD) is the primary modality for evaluating mitral and tricuspid inflow velocities to detect respiratory variations associated with cardiac tamponade physiology. Tissue Doppler imaging (TDI) can also provide complementary information on myocardial motion, highlighting diastolic dysfunction. Clinicians should recognize that pulsus paradoxus, while detectable via echocardiographic findings (e.g., exaggerated inflow variations), is primarily a clinical sign assessed by measuring the inspiratory decline in systolic blood pressure (>10 mmHg) using a sphygmomanometer. Differentiating the clinical sign from echocardiographic findings is vital, as the former reflects systemic hemodynamics and the latter focuses on intracardiac flow dynamics. Despite its utility, Doppler assessment can be technically challenging in unstable or uncooperative patients, particularly if poor acoustic windows or irregular respiratory patterns obscure the findings. In such cases, adjunctive imaging modalities like CT or MRI may provide crucial additional information, especially for evaluating large or loculated pericardial effusions that are difficult to characterize with POCUS alone [2-17].

Clinical Integration of the Findings

Pericardial tamponade presents a spectrum of diseases, ranging from mild elevations in intrapericardial pressure with subtle hemodynamic effects to severe cases causing obstructive shock. Without timely intervention, this pathophysiology can progress to cardiac arrest and potentially result in fatal outcomes. We must take into account the clinical presentation and the various underlying causes, including pericarditis (idiopathic, infective, cancer-related, autoimmune, uremic), medical procedures (e.g., cardiac surgery or catheterization), myocardial infarction, hypothyroidism, trauma, and aortic dissection. The development of tamponade depends on the rate of pericardial fluid accumulation and pericardial compliance, which justifies that we do not assess its risk based only on the size of the pericardial effusion [2-17]. 

Pericardial tamponade occurs when fluid in the pericardial space causes significant hemodynamic instability and symptoms directly related to the effusion. Beck’s triad: hypotension, muffled heart sounds, and jugular venous distention are classically associated with cardiac tamponade. However, all three components are infrequently observed together in clinical practice, with studies suggesting their presence in fewer than 30% of cases. For example, muffled heart sounds may be difficult to detect in noisy or emergent settings, and hypotension may not develop in cases with chronic or slowly accumulating effusions. Clinicians should remain vigilant even when Beck’s triad is incomplete, particularly when other signs, such as pulsus paradoxus or echocardiographic features, are present. This condition develops when the fluid pressure in the pericardial sac exceeds the pressure within the heart chambers, obstructing their normal filling. In cases of small-volume, rapidly accumulating tamponade, such as hemopericardium following trauma, even 100-200 mL of blood can precipitate severe hemodynamic compromise due to the rigid pericardium's inability to stretch quickly. In contrast, with chronic effusions, the pericardium gradually adapts, allowing it to handle larger volumes of fluid (up to two liters) before tamponade symptoms become evident. As the pericardium’s ability to stretch nears its limit, even small additional volumes of fluid can cause significant increases in intrapericardial pressure. These contrasting examples emphasize the importance of clinical context when interpreting imaging findings and determining urgency [2-17].

There are several factors that determine the hemodynamic impact: fluid characteristics (blood vs. serous), anatomical distribution (loculated vs. circumferential), integrity of pericardial layers (inflammation, neoplasia, fibrin), patient volume status, and size, thickness, and function of underlying cardiac cavities. In aortic dissections, PEF can be present if the dissection is retrograde and echogenic. The finding of an intimal flap and dilatation of the aortic root can change the management and confirm probable life-threatening complications. Since only the proximal part of the ascending aorta can be seen, aortic dissection cannot be ruled out completely by this modality of imaging, and angio-CT should be considered in patients with high clinical suspicion. Cardiac MRI, with its superior soft-tissue characterization, excels in differentiating pericardial masses and thickened pericardium, making it useful in subacute or chronic presentations such as neoplastic effusions. In resource-limited settings, the clinical signs can be complemented with rapid diagnosis with focused cardiac ultrasound using portable devices [2-17].

Once the diagnosis of PEF is established and excludes the need for emergent interventions, the fluid characteristics should serve to help identify the type of effusion, which can be crucial for determining the appropriate treatment approach. Unechogenic space is usually associated with serous fluid, while corpusculated or echogenic fluid may indicate hemorrhagic or purulent effusions. Solid masses inside the pericardium may suggest hematomas or neoplastic diseases (Table 4) [2-17].

**Table 4:** Echogenicity and conditions associated.

Echogenicity	Conditions associated
Anechoic fluid (unechogenic)	Commonly seen in simple pericardial effusion, often associated with conditions like viral infections, uremic pericarditis, or idiopathic pericarditis.
Echogenic fluid	Seen in inflammatory effusions such as infectious pericarditis, malignant effusions, or effusions related to trauma (e.g., hemopericardium).
Complex or corpusculated fluid	Typically found in more complicated effusions, such as those associated with tuberculosis, malignancy, or trauma-induced bleeding (hemopericardium).

Proposed algorithmic assessment

**Figure 1:**
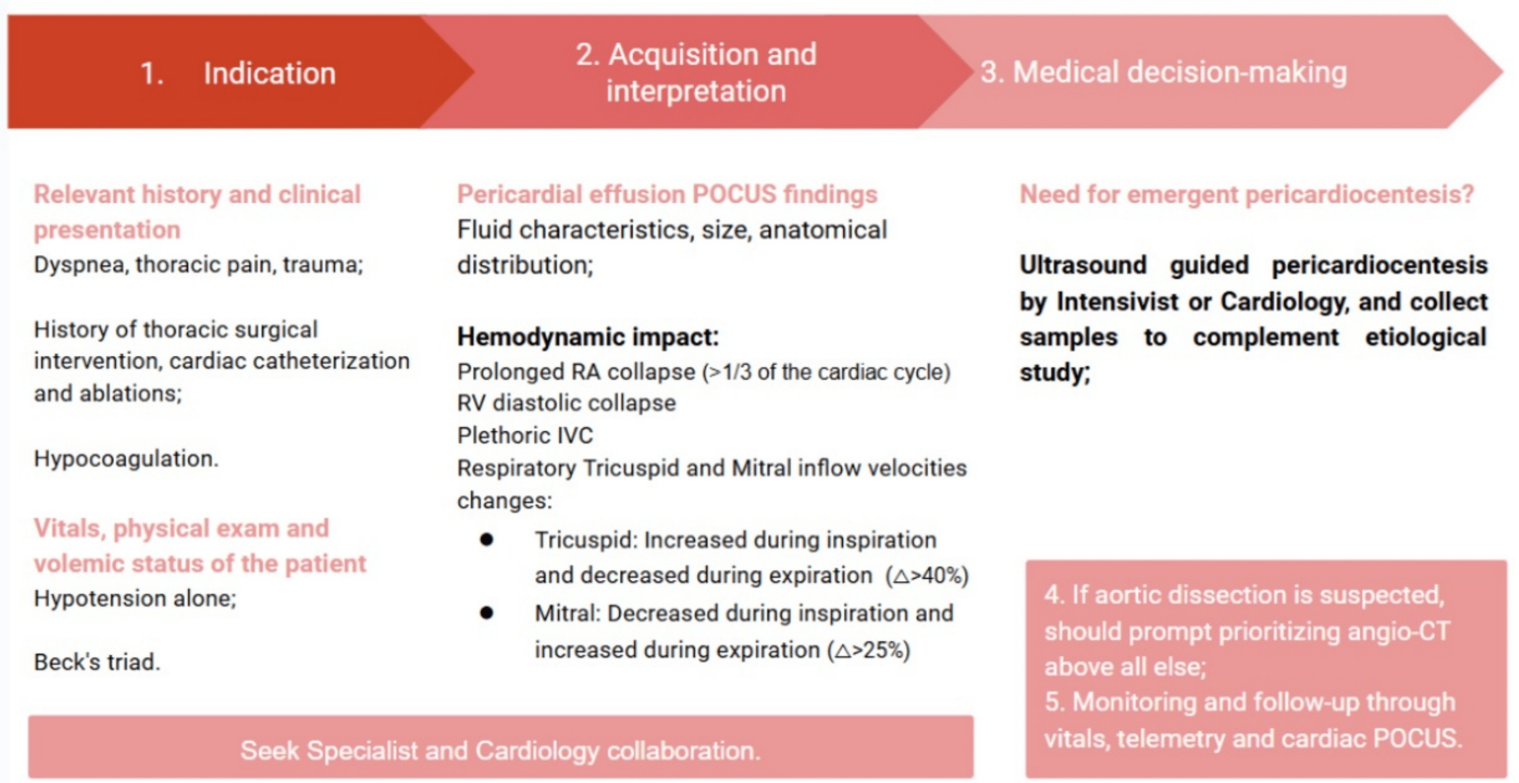
Proposed algorithm for pericardial effusion approach using the I-AIM model. POCUS: point-of-care ultrasound; RA: right atrial; RV: right ventricular; IVC: inferior vena cava Image Credits: Sofia Moura de Azevedo

So, to integrate the PEF finding in a clinical perspective, following the I-AIM model for an habilitated POCUS user, we propose the following algorithmic assessment (Figure 1) [2-19].

## Conclusions

POCUS has markedly enhanced the assessment of PEFs, offering a swift and accurate diagnostic tool at the bedside. This technology enables clinicians to evaluate the effusion's size, detect cardiac tamponade, and guide pericardiocentesis procedures, thereby improving patient outcomes. Proper training and awareness of the limitations of POCUS are critical to ensure accurate assessments and to avoid misdiagnoses that, in a critically ill patient, can result in unnecessary pericardiocentesis, potentially leading to severe complications such as cardiac chamber perforation, worsening tamponade, or even death. Therefore, integrating ultrasound findings with the patient's overall clinical condition, including their symptoms and signs, is vital. In ambiguous cases, it may be beneficial to manage other clearly identified effusions, such as pleural effusion or ascites, before approaching the PEF. If the PEF is small and does not significantly affect the right heart chambers' function, drainage should generally be avoided unless infection is suspected. For complex cases, besides specialist consultation, advanced imaging should be considered. The highlighted recommendations to improve accuracy are standardization of imaging techniques and protocols, confirmation through multiple views, and collaboration between clinicians. Incorporating POCUS into routine practice enhances diagnostic accuracy and enables timely interventions, leading to improved care for patients with PEFs. Future advancements, including artificial intelligence (AI)-driven POCUS interpretation and expanded training, hold promise for enhancing diagnostic accuracy and broadening the tool's applicability across diverse clinical settings.

## References

[1] Ma IW, Arishenkoff S, Wiseman J (2017). Internal medicine point-of-care ultrasound curriculum: consensus recommendations from the Canadian Internal Medicine Ultrasound (CIMUS) group. J Gen Intern Med.

[2] Manson WC, Kirksey M, Boublik J, Wu CL, Haskins SC (2019). Focused assessment with sonography in trauma (FAST) for the regional anesthesiologist and pain specialist. Reg Anesth Pain Med.

[3] Ceriani E, Cogliati C (2016). Update on bedside ultrasound diagnosis of pericardial effusion. Intern Emerg Med.

[4] Alerhand S, Adrian RJ, Long B, Avila J (2022). Pericardial tamponade: a comprehensive emergency medicine and echocardiography review. Am J Emerg Med.

[5] Hoch VC, Abdel-Hamid M, Liu J, Hall AE, Theyyunni N, Fung CM (2022). ED point-of-care ultrasonography is associated with earlier drainage of pericardial effusion: a retrospective cohort study. Am J Emerg Med.

[6] Hoch JM, Abdel-Hamid M, Fung C (2021). 332 point-of-care ultrasound decreases time to intervention for patients with pericardial effusions. Annals of Emergency Medicine.

[7] Alpert EA, Amit U, Guranda L, Mahagna R, Grossman SA, Bentancur A (2017). Emergency department point-of-care ultrasonography improves time to pericardiocentesis for clinically significant effusions. Clin Exp Emerg Med.

[8] Hanson MG, Chan B (2021). The role of point-of-care ultrasound in the diagnosis of pericardial effusion: a single academic center retrospective study. Ultrasound J.

[9] Liu RB, Donroe JH, McNamara RL, Forman HP, Moore CL (2017). The practice and implications of finding fluid during point-of-care ultrasonography: a review. JAMA Intern Med.

[10] Dong M, West FM, Cooper J, Foster J, Davis R (2023). A guide to point of care ultrasound examination of a pericardial effusion. The Medicine Forum.

[11] Alerhand S, Carter JM (2019). What echocardiographic findings suggest a pericardial effusion is causing tamponade?. Am J Emerg Med.

[12] Yetter E, Brazg J, Del Valle D, Mulvey L, Dickman E (2017). Delayed cardiac tamponade: a rare but life-threatening complication of catheter ablation. Am J Emerg Med.

[13] Francispragasam M, Yoo JH, Lam TV, Kim DJ (2017). Diagnosis of a pericardial effusion with a thoracic aortic aneurysm by point-of-care ultrasound. CJEM.

[14] Mok KL (2016). Make it SIMPLE: enhanced shock management by focused cardiac ultrasound. J Intensive Care.

[15] McCanny P, Colreavy F (2017). Echocardiographic approach to cardiac tamponade in critically ill patients. J Crit Care.

[16] Blanco P, Volpicelli G (2016). Common pitfalls in point-of-care ultrasound: a practical guide for emergency and critical care physicians. Crit Ultrasound J.

[17] Clancy DJ, Mclean A, Slama M, Orde SR (2018). Paradoxical septal motion: a diagnostic approach and clinical relevance. Australas J Ultrasound Med.

[18] Ruge M, Marhefka GD (2022). IVC measurement for the noninvasive evaluation of central venous pressure. J Echocardiogr.

[19] Bahner DP, Hughes D, Royall NA (2012). I-AIM: a novel model for teaching and performing focused sonography. J Ultrasound Med.

